# Sex differences in experiences of multiple traumas and mental health problems in the UK Biobank cohort

**DOI:** 10.1007/s00127-021-02092-y

**Published:** 2021-05-10

**Authors:** Emma Yapp, Tom Booth, Katrina Davis, Jonathan Coleman, Louise M. Howard, Gerome Breen, Stephani L. Hatch, Matthew Hotopf, Siân Oram

**Affiliations:** 1 Institute of Psychology, Psychiatry and Neuroscience, King’s College London, London, UK; 2 Department of Psychology, University of Edinburgh, Edinburgh, UK; 3 South London and Maudsley NHS Foundation Trust, NIHR Biomedical Research Centre, De Crespigny Park, Denmark Hill, London, UK; 4 ESRC Centre for Society and Mental Health, King’s College London, London, UK

**Keywords:** Violence, Gender-based violence, Mental health, Women’s health

## Abstract

**Purpose:**

Experiences of reported trauma are common and are associated with a range of mental health problems. Sex differences in how reported traumas are experienced over the life course in relation to mental health require further exploration.

**Methods:**

157,358 participants contributed data for the UK Biobank Mental Health Questionnaire (MHQ). Stratified Latent Class Analysis (LCA) was used to analyse combinations of reported traumatic experiences in males and females separately, and associations with mental health.

**Results:**

In females, five trauma classes were identified: a low-risk class (58.6%), a childhood trauma class (13.5%), an intimate partner violence class (12.9%), a sexual violence class (9.1%), and a high-risk class (5.9%). In males, a three-class solution was preferred: a low-risk class (72.6%), a physical and emotional trauma class (21.9%), and a sexual violence class (5.5%). In comparison to the low-risk class in each sex, all trauma classes were associated with increased odds of current depression, anxiety, and hazardous/harmful alcohol use after adjustment for covariates. The high-risk class in females and the sexual violence class in males produced significantly increased odds for recent psychotic experiences.

**Conclusion:**

There are sex differences in how reported traumatic experiences co-occur across a lifespan, with females at the greatest risk. However, reporting either sexual violence or multiple types of trauma was associated with increased odds of mental health problems for both males and females. Findings emphasise the public mental health importance of identifying and responding to both men and women’s experiences of trauma, including sexual violence.

**Supplementary Information:**

The online version contains supplementary material available at 10.1007/s00127-021-02092-y.

## Introduction

A traumatic event is defined as exposure to actual or threatened death, serious injury or sexual violence through direct experience or witnessing in the Diagnostic and Statistical Manual for Mental Disorders 5th edition. Experiences of both violence and trauma are highly prevalent in the general population. Global estimates indicate that up to 36% of people have experienced childhood maltreatment [1], though there are sex differences: 18% of girls report childhood sexual abuse, compared to 8% of boys [1], and 47% of boys^1^ have been in a physical fight in the past year compared to 26% of girls [2]. Sex differences persist for experiences of violence in adulthood: 26% of women and 15% of men report domestic violence and abuse in adulthood [3]. Certain traumatic experiences are likely to co-occur; for example, people who experience one form of childhood maltreatment are likely to experience another, because they are often living with their perpetrator [4]. This continues across the life course. According to stress proliferation theory [5], there are both direct relationships between traumatic experiences (e.g. types of childhood maltreatment) and indirect ones. For example, being a victim of violence or abuse may contribute to the onset of mental health problems, which in turn renders individuals vulnerable to further violence or abuse [6].

Experiences of violence and reported trauma are associated with mental disorders across the diagnostic spectrum and are highly prevalent among people with psychiatric diagnoses [7]. However, questions remain over how to conceptualise combinations of sex differences in traumatic experiences in relation to mental health. While some research emphasises that mental health service users have experienced multiple forms of abuse [8], there is little consensus over how multiple victimisation differs between the sexes, as this literature typically conducts analyses solely on women [9], or the population as a whole [10]. This gap in the literature raises important questions about how such combinations of experiences differ between the sexes, and how they should be quantified in relation to mental health research and clinical practice.

Previous research has been dominated by additive approaches in which each additional traumatic experience is assumed to increase the odds of developing mental health problems to the same extent. This method has been criticised [11] for its assumption that all traumas contribute equally to the development of mental health problems. Some researchers have therefore begun to use variable-centred approaches, such as factor analysis, to examine how traumatic events group together [12]. Factor analytic methods have also been criticised, as they assume that the studied population all experience traumas in the same ways [13]; studying the “effect”, rather than the “cause” [14].

 Person-centred approaches, such as Latent Class Analysis (LCA), in which the individual is the unit of analysis, offer an alternative to these methods. LCA seeks to identify groups of individuals who report exposure to combinations of trauma. LCA has been applied to diverse arrays of traumatic experiences [13], including both interpersonal and non-interpersonal traumas. These studies tend to establish classes characterised by experiences of domestic and sexual violence in women, and classes of non-interpersonal or non-sexual trauma in men [10]. However, direct comparisons between adult men and women within the same cohort are highly limited, and most findings tend to be established within single-sex samples [10]. Compared to classes characterised by a low risk for traumatic experiences, individuals in groups with a higher risk of traumatic experiences have higher odds of experiencing mental health problems [13].

Experiences of violence and trauma are gendered [15, 16], yet there is a paucity of work examining whether stratification of data by sex/gender leads to different patterns of exposure for men and women, and different impacts on mental health [13]. This study, therefore, uses LCA to examine sex differences in exposure to a wide range of interpersonal and other traumas and in association with mental health problems. It uses data from the UK Biobank Mental Health Questionnaire (MHQ)–a large cohort of more than 150,000 adults in the United Kingdom – on lifetime trauma and adversity, including types of traumas more commonly experienced by women (such as domestic and sexual violence). The inclusion of these items is particularly important, as a measurement of domestic and sexual violence has been limited in a large cohort and psychiatric morbidity surveys, meaning that few datasets permit direct comparison between men and women’s experiences of trauma over the life course in relation to mental health [17]. We aim to establish and characterise the sex differences in experiences of multiple reported traumas in the UK Biobank.

## Methods

### Sample

The UK Biobank is a large population-based cohort study of people recruited via assessment centres across the UK between 2006 and 2010. Postal invitations were sent to 9,238,453 individuals registered with the UK’s National Health Services who were aged 40–69 and lived within 25 miles of one of 22 assessment centres throughout the UK. Overall, 503,317 individuals initially consented to participate in the UK Biobank–a participation rate of 5.5%. There is significant evidence of a “healthy volunteer” bias within the UK Biobank; participants have higher socio-economic status, better education and health compared to the general population. This cohort is therefore not representative of the UK general population, and neither are the data produced by the subset of participants who participated in the MHQ [18]. Individuals who participated in the MHQ were more likely to: be female, have attended assessment centres in Greater London and the South West of England, have higher educational attainment, not currently smoke, have fewer hospital diagnoses, and have a family history of dementia or severe depression compared to those who did not participate [19]. Consent was obtained for future longitudinal follow-up and linkage with routinely collected health-related data. The MHQ was administered online to participants as part of this follow-up in 2016–17 [20]. The MHQ was developed to be short (20–30 min total), with an emphasis on depression and the use of established measures such as the Composite International Diagnostic Interview Short Form (CIDI-SF), and the Childhood Trauma Screener [21]. The MHQ was incorporated into the UK Biobank web platform as a questionnaire about “thoughts and feelings”; a hyperlinked email was then sent to consenting participants who had provided an email address. Of the 339,229 participants invited to complete the MHQ, 157,366 (46.3%) did so [20]. Several individuals subsequently withdrew their consent for their data to be used; 157,358 people are included in our analyses.

### Measures

#### Mental health

Current depression was measured using the Patient Health Questionnaire (PHQ-9); patients who endorsed more than five symptoms as present on “more than half the days” (and “thoughts that you would be better off dead, or of hurting yourself in some way” on at least “several days”) including at least one core symptom (depressed mood and loss of interest) were classified as currently experiencing depression (equivalent to DSM Major Depressive Disorder) [22]. Current anxiety was measured using the seven-item Generalised Anxiety Disorder scale (GAD-7) using the established cut-off point of a score of ten or more [23]. Current hazardous/harmful alcohol use was measured using the Alcohol Use Disorders Identification Test, using the established cut-off: a score of eight or more [24]. Recent psychotic experiences were determined using an abridged version of the psychosis module of the CIDI, determined by whether participants reported psychotic experiences (visual hallucinations; auditory hallucinations; delusions of reference; persecutory delusions) within the last 12 months [25].

#### Trauma and adversity

Adverse childhood experiences were measured using the Childhood Trauma Screener [21], a shortened version of the Childhood Trauma Questionnaire [26]; items asked participants about traumatic experiences while “growing up”; items were endorsed on a five-point Likert scale (“never true”, “rarely true”, “sometimes true”, “often”, “very often true”), and dichotomised. A short checklist with binary answers (“yes, “no”) was used to identify lifetime traumatic experiences as defined by the Diagnostic and Statistical Manual of Mental Disorders [27, 28], and a checklist for measuring adverse experiences (e.g. intimate partner violence) occurring in adulthood (age 16 years and above) was devised by the study team; items on intimate partner violence (IPV) were adapted from the Crime Survey for England and Wales [29]. Two additional items about relationship and financial insecurity were included such that the structure of this questionnaire would be comparable to the Childhood Trauma Screener. For all items relating to experiences of trauma, participants were provided with the opportunity to decline to answer. Altogether, these measures produced 16 items (see Table 1).

**Table 1:** Prevalence of individual traumatic experiences

Item	Question (and item coding)	Whole sample (%)	Missing (%)	Males (%)	Missing (%)	Females (%)	Missing (%)
Childhood trauma screener							
Emotional childhood neglect	When I was growing up I felt loved (less than “often”)	22.4	0.4	21.6	0.4	23.0	0.4
Emotional childhood abuse	When I was growing up people in my family hit me so hard that it left me with bruises or marks (more than “never”)	15.6	0.3	12.7	0.2	17.8	0.3
Physical childhood abuse	When I was growing up I felt like someone in my family hated me (more than “never”)	18.9	0.2	21.1	0.2	17.3	0.3
Physical childhood neglect	When I was growing up someone molested me sexually (more than “never”)	16.3	0.7	15.2	0.6	17.2	0.8
Sexual childhood abuse	When I was growing up there was someone to take me to the doctor if I needed it (less than “very often”)	8.7	1.2	5.8	0.6	10.9	1.6
Adulthood (≥ 16 years) questionnaire							
Relationship insecurity	Since I was sixteen, I have been in a confiding relationship (less than “often”)	31.2	2.6	30.8	2.0	31.6	3.1
Physical intimate partner violence	Since I was sixteen, a partner or ex-partner deliberately hit me or used violence in any other way (more than “never”)	12.8	0.3	8.0	0.2	16.4	0.4
Psychological intimate partner violence	Since I was sixteen, a partner or ex-partner repeatedly belittled me to the extent that I felt worthless (more than “never”)	24.0	0.3	15.7	0.2	30.2	0.4
Sexual intimate partner violence	Since I was sixteen, a partner or ex-partner sexually interfered with me, or forced me to have sex against my wishes (more than “never”)	5.8	0.3	0.8	0.2	9.5	0.4
Financial insecurity	Since I was sixteen, there was money to pay the rent or mortgage when I needed it (less than “very often”)	14.5	1.6	12.8	1.5	15.8	1.6
Lifetime traumatic experiences (childhood or adulthood)							
Serious accident	In your life have you been in a serious accident that you believed to be life-threatening at the time (“yes”)	9.8	0.1	13.5	0.1	7.0	0.1
Witness death	In your life have you witnessed a sudden violent death (e.g. murder, suicide, aftermath of an accident) (“yes”)	13.5	0.2	19.3	0.2	9.0	0.2
Serious illness	In your life have you been diagnosed with a life-threatening illness (“yes”)	16.3	0.4	17.8	0.3	15.1	0.5
Experienced war	In your life have you been involved in combat or exposed to a war-zone (either in the military or as a civilian) (“yes”)	3.6	0.1	6.2	0.2	1.6	0.1
Sexual violence ever	In your life have you been a victim of a sexual assault, whether by a stranger or someone you knew (“yes”)	14.8	1.3	7.6	0.6	20.3	1.8
Physically violent crime	In your life have you been attacked, mugged, robbed, or been the victim of a physically violent crime (“yes”)	19.0	0.2	25.0	0.1	19.0	0.2

#### Socio-demographics and socio-economics

The socio-demographic indicators used in the analysis included: sex, age, ethnicity, migrant status, loneliness, and social isolation. Ethnicity and sex were self-reported, and the ethnicity variable indicated identification with either: Black (Caribbean, African, any other Black background), Asian (Indian, Pakistani, Bangladeshi, any other Asian background), Chinese, Mixed (White and Black Caribbean, White and Black African, any other Mixed background), Other, or White ethnicities (White British, White Irish, any other White background); on account of the low prevalence of non-White ethnicities, ethnicity was coded as a binary variable. Migrant status was determined by whether participants were born in the UK, and loneliness was self-reported. Social isolation was a binary variable determined by participants’ social and community activities; participants were deemed isolated if they were living alone, were visited by family and/or friends less than once a week, and did not attend any regular social activities. The socio-economic indicators used included: Townsend Deprivation Score, education, and household income. Townsend Deprivation Material Scores were based on census data; each participant was assigned a score corresponding to the output area of their residential postcode (least deprived < − 2.00, average − 2.00 to 1.99, or most deprived: ≥ 2.00) [18]. Educational attainment was classified by qualification. Participants reporting degree level; A Levels or equivalent; or secondary school or equivalent (including O levels, CSEs, HNDs, HNCs and NVQs) qualifications were classified as such. Participants reporting none of the above, but the attainment of “other” qualifications (e.g. nursing, teaching) were classified as “other”; and those reporting none of the above were categorised as such. Household income was classified as: under £18,000; £18,000–£30,000; £30,000–£52,000; £52,000–£100,000; and more than £100,000.

#### Statistical analysis

The prevalence of the 16 individual adverse life experiences was calculated both in the overall sample and for males and females separately. Latent class structures were explored in both males and females individually and the sample as a whole, to ensure that the sex-stratified LCA provided the best solution [30].

The fit of up to six models (one-class through to six) was assessed, as a systematic review of person-centred analyses of traumatic items indicated that all included studies identified solutions in this range [13]. Models were evaluated using the Bayesian Information Criterion (BIC) [31], the sample size adjusted BIC (ssaBIC) [32], Pearson’s likelihood ratio chi-square (G^2^) statistic [33], the consistent Akaike Information Criterion (cAIC) [34], and the identification of coherent and conceptually meaningful solutions. Goodness of fit in LCA models is indicated by lower values for cAIC, BIC, and ssaBIC. The accuracy of classification of classes was determined by inspection of average posterior probabilities of class membership (provided in Tables S1 and S2), and entropy measures, with higher values (ranging from 0 to 1) indicating better classification [35]. Thirty random sets of starting values were used to avoid converging on local maxima [33].

Final class solutions were then used to calculate individuals’ class membership using modal class assignment; that is, assigning individuals to the class for which the estimated probability is the largest [30]. Class solutions were regressed on all covariates, to explore the characteristics of individuals in each class; latent class variables were dummy coded for both males and females. Current or recent mental health problems were regressed on class membership using logistic regressions; both adjusted and unadjusted models are presented. Adjusted regression models controlled for age, ethnicity (White vs non), Townsend Deprivation Score, educational attainment, household income, migrant status, loneliness, and social isolation. All analyses were conducted in R statistical computing environment [36] version 3.5.1, and LCA was conducted using the poLCA package [33]. Missing data were handled using available case analysis, apart from during the LCA, as poLCA can accommodate missing values by initially excluding missing data on any indicator variables to calculate prior probabilities, and then updating these probabilities using as many indicator variables as are observed for each individual after calculating posteriors [33].

## Results

Data from 157,358 participants were included in our analyses: 68,261 males and 89,097 females (see Table S3). The largest proportion of participants in both sexes were aged 65–74 (42.5% females, 47.2% males), of White ethnicity (96.7% females, 96.6% males), living in areas characterised as least deprived by the Townsend Deprivation Index (55.6% females, 57.4% males), and earning £30,000–£52,000 as a household (24.9% females, 27.4% males). Proportions were consistent across the sexes, and the demographic details of this sample have been discussed in detail elsewhere [20].

### Prevalence of trauma and adversity

The prevalence of reported trauma and adversity is presented in Table 1. For both males and females, the most common adverse experience was relationship insecurity in adulthood (≥ 16years), reported by 30.8% and 31.6%, respectively. The second most common adverse experience was, for males, physically violent crime ever (25.0%) and for females, psychological abuse from an intimate partner in adulthood (31.2%). The prevalence rates for sexual childhood abuse, sexual intimate partner violence, and sexual violence ever all differ. Although there is the conceptual overlap between these items, overlap between positive responses was relatively low, and thus these items are treated as independent from each other in ensuing analyses. Missing data were between 0.1 and 3.1% across items.

### Determining latent classes

Table 2 presents the goodness of fit statistics for sex-stratified latent class models. The optimum solution for the whole sample was determined as the four-class solution (see Table S4), yet optimum class solutions differed within males and females separately, and thus results are reported for these groups individually only.

**Table 2:** Fit statistics for sex-stratified latent class analysis

Females						Males					
Classes	BIC^a^	ssaBIC^b^	cAIC^c^	Likelihood ratio	Entropy	Classes	BIC	ssaBIC	cAIC	Likelihood ratio	Entropy
1	1,186,015.7	1,185,964.9	1,186,031.7	133,026.7	–	1	850,853.9	850,803.1	850,869.9	72,922.3	–
2	1,112,505.9	1,112,401.0	1,112,538.9	66,113.2	0.700	2	818,783.9	818,679.0	818,816.9	43,228.8	0.607
3	1,099,683.0	1,099,524.1	1,099,733.0	54,427.0	0.716	**3**	**811,849**.5	**811,690**.6	**811,899**.5	**36,360**.**0**	**0**.**654**
4	1,090,151.9	1,089,939.0	1,090,218.9	45,301.6	0.734	4	805,420.8	805,207.8	805,487.8	30,201.8	0.646
**5**	**1,081,392**.**6**	**1,081,125**.**7**	**1,081,476**.**6**	**37,296**.**4**	**0**.**708**	5	801,520.1	801,253.2	801,604.1	26,460.6	0.602
6	1,075,009.1	1,074,688.1	1,075,110.1	31,199.2	0.692	6	798,850.6	7,982,529.7	798,951.6	23,703.2	0.623

Optimum class solutions in males and females separately are highlighted in bold

^a^Bayesian information criterion

^b^Sample size adjusted Bayesian Information Criterion

^c^Consistent Akaike Information Criterion

In females, the BIC, ssaBIC, and cAIC all decreased with the introduction of additional classes. However, the entropy was highest for the four-class model; the four and five class models were, therefore, considered the best candidates for the optimal solution, and the average posterior probabilities were relatively comparable across both solutions (see Table S2), but the five class solution was preferred on the basis of its conceptual coherence (see Figs. S1 and S2); model-predicted probabilities are displayed in Table 3. Class 1 (*N* = 10,533, 12.9%) indicated high probabilities of items relating to physical and psychological intimate partner violence and was thus labelled the *intimate partner violence class.* Class 2 (*N* = 55,063, 58.6%) indicated low probabilities across all adverse items and was labelled the *low-risk class*. Class 3 (*N* = 5051, 5.9%) was characterised by generally high probabilities across adverse items and was labelled the *high-risk class*. Class 4 (*N* = 7644, 9.1%) exhibited high probabilities of the sexual violence and abuse items (sexual assault ever and childhood sexual abuse) and was labelled the *sexual violence class.* Class 5 (*N* = 10,806, 13.5%) was characterised by relatively high probabilities of experiencing physical abuse and emotional abuse and neglect in childhood, plus relationship insecurity in adulthood; this was labelled the *childhood trauma class.*

**Table 3:** Model-predicted probabilities for 3 class solution in males and 5 class solution in females

	Males			Females				
	Low risk	Physical and emotional trauma	Sexual violence	Low risk	Sexual violence	Intimate partner violence	Child abuse	High risk
Childhood trauma and adversity								
Emotional childhood neglect	0.09	0.56	0.39	0.05	0.29	0.17	0.75	0.85
Physical childhood abuse	0.11	0.51	0.42	0.05	0.27	0.16	0.41	0.69
Emotional childhood abuse	0.03	0.42	0.29	0.04	0.24	0.14	0.53	0.80
Sexual childhood abuse	0.01	0.03	0.76	0.01	0.63	0.08	0.05	0.49
Physical childhood neglect	0.10	0.31	0.22	0.10	0.16	0.11	0.39	0.54
Adulthood (≥ 16 years) trauma and adversity								
Relationship insecurity	0.26	0.49	0.35	0.24	0.28	0.38	0.52	0.65
Physical intimate partner violence	0.03	0.21	0.19	0.03	0.09	0.68	0.09	0.68
Psychological intimate partner violence	0.08	0.37	0.31	0.12	0.23	0.91	0.32	0.87
Sexual intimate partner violence	0.00	0.02	0.05	0.01	0.08	0.37	0.03	0.53
Financial insecurity	0.09	0.25	0.16	0.10	0.12	0.26	0.23	0.45
Lifetime traumatic experiences (childhood or adulthood)								
Serious accident	0.10	0.24	0.23	0.04	0.12	0.10	0.07	0.18
Witness death	0.15	0.30	0.30	0.06	0.15	0.11	0.09	0.21
Serious illness	0.16	0.21	0.24	0.13	0.17	0.17	0.17	0.22
Experienced war	0.04	0.11	0.11	0.01	0.03	0.02	0.02	0.04
Sexual violence ever	0.02	0.04	0.94	0.05	0.92	0.30	0.08	0.75
Physically violent crime	0.19	0.40	0.43	0.10	0.23	0.21	0.14	0.37

In males, the BIC, ssaBIC, and cAIC similarly all decreased with the addition of more classes. However, the entropy measure indicated that the best candidates for the optimal model were the three and four class models. The three-class model was again chosen on the basis of conceptual coherence (see Figs. S3 and S4) and displayed marginally more consistent average posterior probabilities than the four-class solution (see Table S3); model-predicted probabilities are displayed in Table 3. Class 1 (*N* = 3589, 5.5%) was characterised by moderate strength probabilities across most items, with a particularly high probability of sexual violence ever and childhood sexual abuse; this was labelled the *sexual violence class.* Class 2 (*N* = 13,311, 21.9%) exhibited medium-strength probabilities across several items and a low probability of sexual violence; this was labelled the *physical and emotional* trauma class. Class 3 (*N* = 51,361, 72.6%) displayed low probabilities across all adverse items and was labelled the *low-risk class.*

### Associations of trauma classes with socio-demographic and socio-economic variables

Class solutions were dummy coded using the low-risk category as the reference category for both sexes. Logistic regressions were used to investigate the socio-demographic and socio-economic characteristics of individuals in each class.

Compared to the low-risk group, females in lower household income brackets, living in more deprived TDI areas, and who were classified as lonely were more likely to be members of trauma classes (see Table 4); females who identified as non-White were more likely to be members of the sexual violence, childhood trauma, and high-risk groups; females born outside of the UK had increased odds of being in the childhood trauma group; and females who were socially isolated were more likely to be members of the intimate partner violence, childhood trauma, and high-risk groups. The sexual violence and intimate partner violence classes were characterised by marginally higher education compared to the low-risk group, whereas the childhood trauma class reflected lower education compared to low risk, although the effect sizes were extremely small. Compared to the low-risk group, males who were in lower household income brackets, living in more deprived areas, who were lonely, and who were socially isolated were more likely to be members of the trauma classes. Males who identified as non-White, and who were born outside of the UK were additionally more likely to be in the sexual violence class compared to the low-risk class. Males with higher educational status were less likely to be in the class characterised by sexual violence.

**Table 4:** Associations between socio-demographic and socio-economic variables and trauma classes compared to the low risk class

	Females				Males	
	Sexual violence vs Low risk: OR (95% CI)	Intimate partner violence vs Low risk: OR (95% CI)	Childhood trauma vs Low risk: OR (95% CI)	High risk vs Low risk: OR (95% CI)	Physical and emotional trauma vs Low risk: OR (95% CI)	Sexual violence vs Low risk: OR (95% CI)
Age	1.0 (1.0–1.0)	1.0 (1.0–1.0)	1.0 (1.0–1.0)	0.9 (0.9–1.0)	1.0 (1.0–1.0)	1.0 (1.0–1.0)
Ethnicity^a^	1.4 (1.2–1.6)	1.1 (0.9–1.3)	1.8 (1.5–2.0)	2.1 (1.8–2.5)	1.6 (1.4–1.8)	1.3 (1.1–1.7)
Migrant status^b^	1.2 (1.0–1.3)	1.0 (0.9–1.1)	1.2 (1.1–1.3)	1.1 (1.0–1.2)	1.2 (1.1–1.3)	1.2 (1.0–1.4)
TDI^c^						
Average	1.3 (1.2–1.3)	1.3 (1.3–1.4)	1.2 (1.1–1.2)	1.5 (1.4–1.7)	1.3 (1.2–1.3)	1.4 (1.3–1.5)
Most deprived	1.6 (1.5–1.7)	1.8 (1.6–1.9)	1.3 (1.2–1.4)	2.4 (2.2–2.6)	1.6 (1.5–1.7)	2.1 (1.9–2.3)
Education^d^						
Other	1.4 (1.2–1.7)	1.1 (0.9–1.2)	1.0 (0.9–1.1)	1.0 (0.8–1.2)	0.9 (0.8–1.0)	1.4 (1.1–1.7)
GCSE	1.2 (1.1–1.4)	1.3 (1.2–1.5)	0.9 (0.8–1.0)	1.1 (0.9–1.3)	0.9 (0.8–0.9)	1.0 (0.9–1.2)
A Level	1.3 (1.1–1.5)	1.4 (1.2–1.6)	0.9 (0.8–0.9)	1.1 (0.9–1.3)	0.9 (0.8–0.9)	1.1 (0.9–1.3)
Degree level	1.5 (1.3–1.7)	1.3 (1.2–1.5)	0.9 (0.8–1.0)	1.2 (1.0–1.4)	0.7 (0.7–0.8)	1.1 (0.9–1.2)
Household income						
£18,000–£30,000^e^	0.9 (0.8–1.0)	0.7 (0.7–0.8)	0.8 (0.8–0.9)	0.6 (0.5–0.6)	0.8 (0.7–0.8)	0.8 (0.7–1.0)
£30,000–£52,000	0.9 (0.8–1.0)	0.6 (0.6–0.7)	0.8 (0.7–0.8)	0.4 (0.4–0.5)	0.8 (0.7–0.8)	0.8 (0.7–0.9)
£52,000–£100,000	0.9 (0.8–0.9)	0.5 (0.5–0.6)	0.7 (0.6–0.7)	0.3 (0.3–0.4)	0.7 (0.6–0.7)	0.8 (0.7–0.9)
Over £100,000	0.9 (0.8–1.0)	0.5 (0.4–0.5)	0.6 (0.5–0.6)	0.3 (0.3–0.3)	0.6 (0.6–0.7)	0.8 (0.7–1.0)
Loneliness^f^	1.5 (1.3–1.8)	1.8 (1.6–2.0)	2.7 (2.5–3.0)	3.6 (3.1–4.1)	2.3 (2.1–2.5)	1.9 (1.7–2.3)
Social isolation^g^	1.1 (1.0–1.2)	1.2 (1.1–1.3)	1.5 (1.4–1.6)	1.6 (1.5–1.8)	1.4 (1.3–1.5)	1.3 (1.1–1.4)
Model N	52,675	55,152	55,081	50,351	57,658	59,124

^a^Ethnicity was coded as a binary variable: 0 = White, 1 = non-White

^b^Migrant status was coded as a binary variable: 0 = non-migrant, 1 = migrant

^c^Tertiles were derived from postcode areas; categories used least deprived TDI as the reference category

^d^Each education level used “none of the above” as the reference category

^e^All household income categories used under £18,000 as the reference category

^f^Self-reported

^g^Determined by participants’ self-reported social and community activities, and whether they lived alone; coded as a binary variable 0

### Associations with current or recent mental health

As shown in Table 5, in females, membership of trauma classes was significantly associated with depression, anxiety, and hazardous/harmful alcohol use compared to the low-risk class in both unadjusted and adjusted analyses. Only the high-risk class was associated with psychotic experiences. In males, both classes were associated with depression, anxiety, and hazardous/harmful alcohol use compared to the low-risk class in both unadjusted and adjusted analyses. In adjusted analyses, only the sexual violence class was associated with psychotic experiences.

**Table 5:** Unadjusted and adjusted odds ratios (ORs) and confidence intervals for current and recent mental health outcomes by latent class compared to the low-risk class

	Unadj	Adj^a^	Unadj	Adj	Unadj	Adj	Unadj	Adj
	Depression		Anxiety		Alcohol		Psychotic experiences	
Females								
Sexual violence	3.1 (2.6–3.7)	2.5 (2.0–3.0)	2.7 (2.2–3.2)	2.4 (2.0–2.9)	1.8 (1.7–1.9)	1.6 (1.5–1.7)	1.3 (0.9–1.8)	1.1 (0.8–1.6)
Intimate partner violence	4.0 (3.5–4.6)	2.9 (2.5–3.4)	3.2 (2.8–3.8)	2.7 (2.3–3.2)	1.9 (1.8–2.0)	1.8 (1.6–1.9)	1.1 (0.8–1.6)	1.0 (0.7–1.4)
Childhood trauma	4.8 (4.2–5.5)	3.8 (3.3–4.5)	4.7 (4.1–5.3)	4.0 (3.4–4.6)	1.3 (1.2–1.4)	1.3 (1.2–1.4)	1.4 (1.0–1.9)	1.2 (0.8–1.7)
High risk	14.8 (12.9–16.8)	8.9 (7.6–10.4)	14.1 (12.4–16.2)	9.2 (7.9–10.9)	2.0 (1.8–2.1)	1.9 (1.7–2.1)	2.8 (2.1–3.6)	2.0 (1.5–2.7)
Males								
Physical and emotional trauma	5.9 (5.1–6.7)	4.1 (3.5–4.7)	5.4 (4.7–6.2)	4.0 (3.4–4.7)	1.3 (1.3–1.4)	1.3 (1.3–1.4)	1.5 (1.2–1.9)	1.2 (1.0–1.7)
Sexual violence	6.5 (5.4–7.8)	5.0 (4.0–6.1)	6.0 (4.9–7.4)	4.8 (3.8–6.0)	1.4 (1.3–1.6)	1.4 (1.3–1.6)	2.2 (1.6–3.0)	1.9 (1.4–2.6)

^a^Odds ratios adjusted for: age, ethnicity, Townsend Deprivation Index, education, household income, migrant status, loneliness, and isolation

## Discussion

This study constitutes one of the largest latent class analyses (LCA) identifying how combinations of interpersonal and non-interpersonal trauma are experienced by males and females over the life course. Our analysis demonstrates differences in the patterning of reported traumatic experiences between males and females, and shows that more females were members of classes characterised by multiple traumas (41%) than were males (27%). Among both males and females, membership of classes characterised by multiple traumas was associated with increased odds of mental health problems, with the highest odds among females in the high-risk group.

The prevalence of individual adverse life events was consistent with other large samples and population-based data, for example the Adverse Childhood Experiences (ACE) study [4] (as well as child maltreatment research conducted more recently in the UK context [37, 38]), the Crime Survey for England and Wales [39], and the World Mental Health surveys [40]. This is despite the UK Biobank MHQ participants being unrepresentative of the general population (being predominantly White, having higher socio-economic status, better education, and better health than the UK average) [18]. Higher prevalence of psychological intimate partner violence was found for the UK Biobank MHQ (30% in females, 16% in males) when compared to surveys conducted with similar questions in England and Wales [41] (18% in females, 8% in males); these may be attributable to differences in questionnaire response options [42].

Our analysis grouped male participants into a low-risk class, a physical and emotional trauma class, and a sexual violence class. Notably, our study established a class of males characterised by sexual violence, the trauma profile of which differed from that of the female sexual violence class, as it was characterised by additional moderate risks of physical victimisation in childhood and adulthood; a similar finding was recently established in a large (*N* = 34,653) sex-stratified LCA of childhood maltreatment [43]. This group of males may have been obscured in non-stratified LCAs [13]. While experiences of sexual violence are more prevalent in females, little exploration has been previously conducted of how male experiences of sexual violence cluster with other traumas. An estimated 3% of males report sexual violence before the age of 16 in England and Wales [37], and 4% thereafter [44]. Males who are sexually victimised in childhood are also significantly more likely to be physically and sexually victimised in adulthood [45]. The male sexual violence class identified by our analysis experienced increased odds of all current or recent mental health problems; a finding also established in the aforementioned stratified LCA of child maltreatment [43]. This group is worthy of further investigation, in terms of understanding male experiences of sexual violence in combination with other traumas, causal pathways and moderators of mental health outcomes, and investigation of optimal service responses and therapeutic interventions.

Females were grouped into five classes: a low-risk class, a sexual violence class, an intimate partner violence class, a childhood trauma class, and a high-risk class; similar findings have been established previously [9]. While previous research conducted in the UK context has and shown that women were more likely to be members of classes characterised by a high risk of violence and abuse [46], our analysis indicates that the high-risk class in this cohort is unique to females. While the male sexual violence class had increased risk of physical victimisation, the high-risk class indicated that 5.9% females multiply experience childhood trauma, sexual violence, and partner violence.

Being a member of all classes characterised by reporting trauma increased a person’s odds of currently experiencing depression, anxiety, and hazardous/harmful alcohol use compared to the low-risk classes in this analysis. These findings fit with other psychiatric literature demonstrating high rates of adverse life experiences among people experiencing anxiety and depression [7], as well as research scrutinising the relationship between adverse life experiences and alcohol use [47]. The high-risk class in females had the strongest associations with all recent mental health problems, and only the high-risk class in females and the sexual violence class in males were associated with recent psychotic experiences. This finding contrasts other work that shows associations between experiences of childhood trauma [48], sexual violence [9, 10], non-interpersonal traumas [10] and psychotic experiences. This may be due to the unrepresentativeness of both the UK Biobank and the constituent MHQ [18, 19], as the recruitment strategies for this cohort may have been likely to exclude people with recent psychotic experiences. Nevertheless, our findings underscore the enduring psychiatric impact of multiple traumas including sexual violence and have important implications.

### Implications

Our findings demonstrate that there are distinct classes of males and females experiencing violence and trauma, and that members of these classes experience increased odds of mental health problems. Early identification of experiences of violence and abuse in health services may help mitigate the enduring effects of violence. In mental health services, recent policy and guidance have largely focused on identifying experiences of childhood sexual abuse [49] and domestic violence and abuse [50] on account of their high prevalence amongst mental health service users [8, 51, 52]. Our findings emphasise the importance of identifying other experiences of trauma; in particular, experiences of multiple victimisation and sexual violence. Referrals and clinical pathways differ across experiences of trauma and violence, and identification methods are often focused on particular cohorts of people [50]. People who use mental health services often complain of traumatic experiences not being identified sooner, and the impact of this on treatment [53]. Taking a universal approach to experiences of adversity amongst mental health service users may therefore enable earlier identification of trauma, and consequently improve treatment outcomes. Although a significantly greater proportion of females are subjected to sexual violence than males in the general population [44], experiences of sexual violence are highly prevalent among both male and female mental health service users [51]. Despite this, mental health services rarely conduct routine enquiry into adulthood sexual violence [54]. Enquiry into childhood physical abuse may be similarly productive, as this too is under-identified by mental health services [52], and our findings indicate a small group of males who experience both childhood physical abuse and lifetime sexual violence.

### Strengths and limitations

To our knowledge, this is the largest study to conduct a sex-stratified latent class analysis on experiences of violence and trauma across the life course and to analyse the relationship between class membership and current or recent mental health. This cohort provided rich data on trauma experienced from childhood to older adulthood, as well as robust measures of mental health, and a diverse array of sociodemographic and socioeconomic variables, that allowed us to interrogate how trauma exposure is shaped by life experience.

There are several important limitations to note. Participants in the UK Biobank and MHQ are not representative of the general population [18, 19]. The majority of participants were White, and recruitment to the cohort was consistent with the ‘healthy volunteer’ effect, such that participants tended to be in better health than the general population [18]. However, generalisable associations with risk factors can be obtained in non-representative samples such as these, provided sufficiently large numbers of people with a range of exposures are included [55]. In addition, many of the items used to assess traumatic experiences may suffer from recall bias, and had either not been validated in this population, or were scored using different methods elsewhere in the literature [21]. In addition, the questions about relationship and financial security may have been interpreted in such a way that they are not indicative of trauma. The measurement of sex was binary and precluded the assessment of gendered experiences of trauma. There is also an issue of temporality, as many of the lifetime adverse experiences either overlap with each other or do not specify when the trauma occurred.

While the entropy measures indicated moderate separation between classes, these statistics were lower than some other work using latent class analysis to analyse traumatic experiences [56]. This is likely because other research has focused on more homogeneous groups of traumatic items–for example, conducting LCA of childhood maltreatment [43, 56], as types of childhood maltreatment often co-occur [4]. The heterogeneity of traumatic experiences studied in our research will bear on class separation, as we examined both interpersonal and non-interpersonal traumas; our entropy measures were comparable to other work examining similar ranges of traumatic experiences [13].

It should also be noted that the traumatic items represented in the UK Biobank MHQ cohort, and therefore in this analysis, are by no means exhaustive. Members of the team involved in developing the MHQ had an interest in experiences of violence against women, including domestic and sexual violence (authors SO and LMH). Several trauma items included in the MHQ measured experiences of violence that are more prevalent in women than men, and this is reflected in the class solutions identified; had the MHQ focused on traumatic experiences more commonly experienced by men, the class solutions would likely have differed. For example, other studies have asked more detailed questions probing experiences of threats, physical violence, and the witnessing of violence or death, all of which were more likely to be experienced by men [57]. These items, as well as ones pertaining to the death of loved ones, and the witnessing of trauma, should be investigated further with regard to associations with mental health. Experiences of trauma and violence may further vary across countries [58]. The groups of latent classes identified here may therefore not be representative of the general population, but rather represent groups of individuals specific to this sample, in this country. The analysis of associations between socio-demographic variables and class membership, therefore, provides important information about who is in each class, and this should be taken into account when interpreting the findings.

### Conclusions

This study established that males and females in the UK Biobank MHQ Cohort experience different patterns of trauma and adversity over their lifetime, with females more likely to experience multiple types of trauma. Among both males and females, experiencing multiple types of trauma was associated with current and recent mental health problems, with odds highest among classes characterised by multiple victimisation and sexual violence. Longitudinal and sex-disaggregated evidence is needed to further unpack the relationship between experiences of trauma, adversity, and mental health over the life course among males and females.

### Supplementary Information

Below is the link to the electronic supplementary material.

Supplementary file1 (DOCX 195 KB)

Supplementary file2 (DOCX 187 KB)

Supplementary file3 (DOCX 187 KB)

Supplementary file4 (DOCX 179 KB)

Supplementary file5 (DOCX 14 KB)

Supplementary file6 (DOCX 13 KB)

Supplementary file7 (DOCX 15 KB)

Supplementary file8 (DOCX 14 KB)

## Data Availability

UK Biobank data is open-access subject to the usual access procedures (www.ukbiobank.ac.uk); the data used in this analysis are from an approved extension to application 16,577 (G. Breen).

## Footnotes

1 Herein, we use “sex” to refer to the sex assigned to people at birth, and “gender” to describe self-identification as a man, woman, or non-binary/genderqueer person. This discussion is inherently limited by the literature, which frequently subscribes to binary representations (and conflations) of both sex and gender. The content of this paper focuses on sex as a binary variable, as this was the only measure used in the UK Biobank data.

## Code availability

R code is freely available on the King’s College London data repository: 10.18742/RDM01-727.
