# Supplementary figures and images for: Sex differences in experiences of multiple traumas and mental health problems in the UK Biobank cohort
Source: Soc Psychiatry Psychiatr Epidemiol. 2021 May 10;58(12):1819–31. doi: 10.1007/s00127-021-02092-y (PMC10628045; doi:10.1007/s00127-021-02092-y)

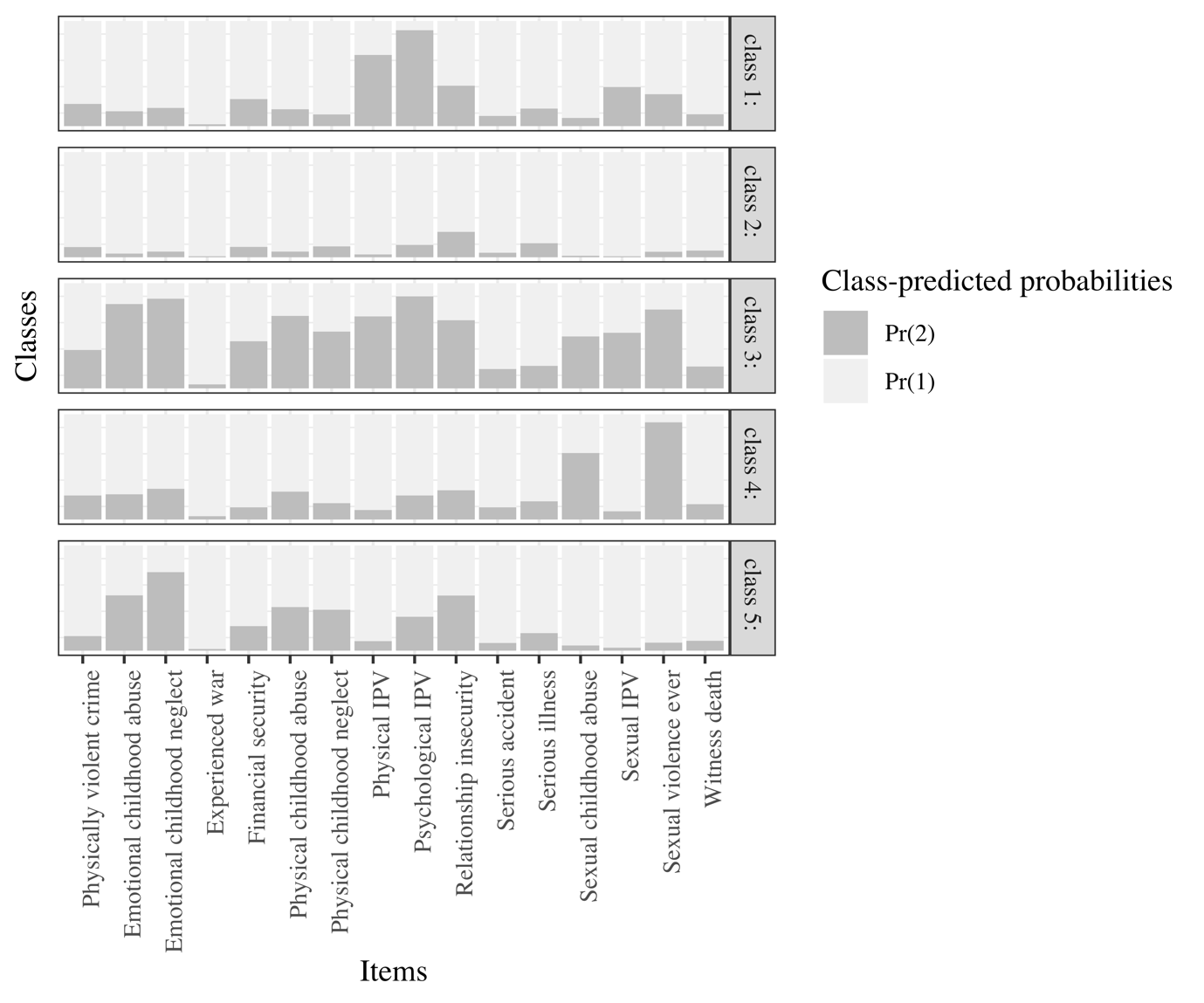


**Figure S1.** 5 class solution in females.

Supplement: Supplementary file 1 — Supplementary file1 (DOCX 195 KB) [file 127_2021_2092_MOESM1_ESM.docx]

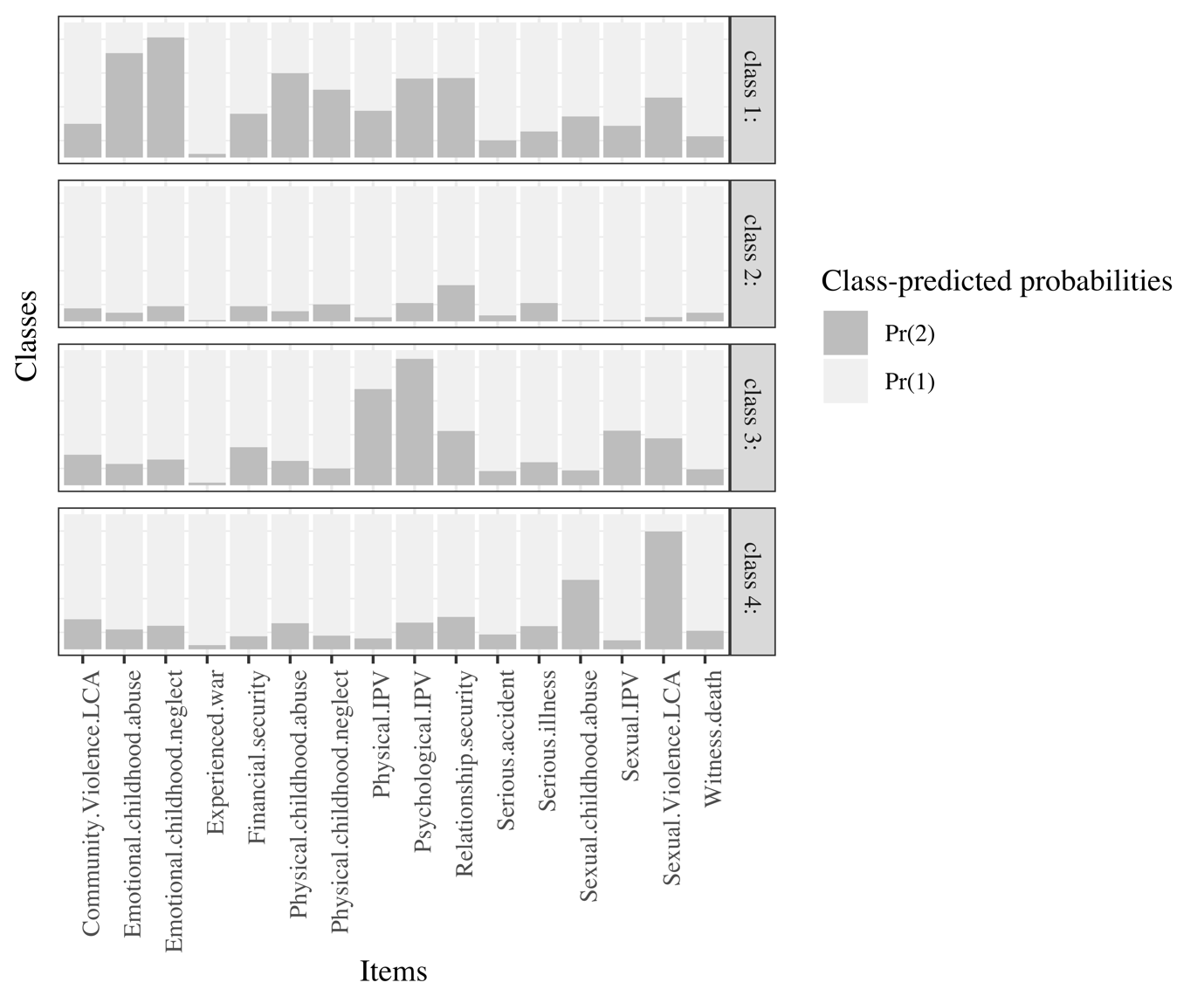


**Figure S2.** 4 class solution in females.

Supplement: Supplementary file 2 — Supplementary file2 (DOCX 187 KB) [file 127_2021_2092_MOESM2_ESM.docx]

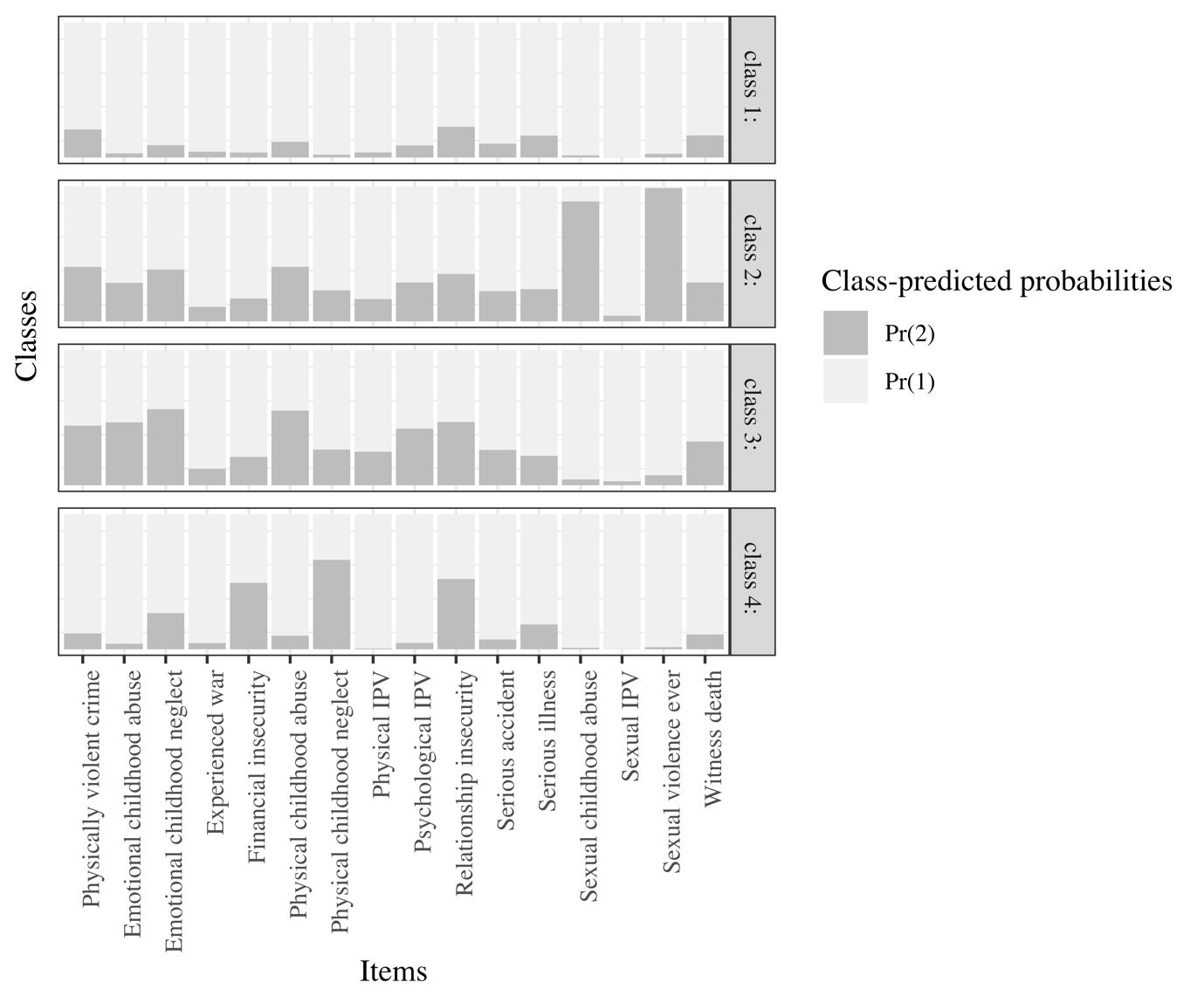


**Figure S3.** 4 class solution males.

Supplement: Supplementary file 3 — Supplementary file3 (DOCX 187 KB) [file 127_2021_2092_MOESM3_ESM.docx]

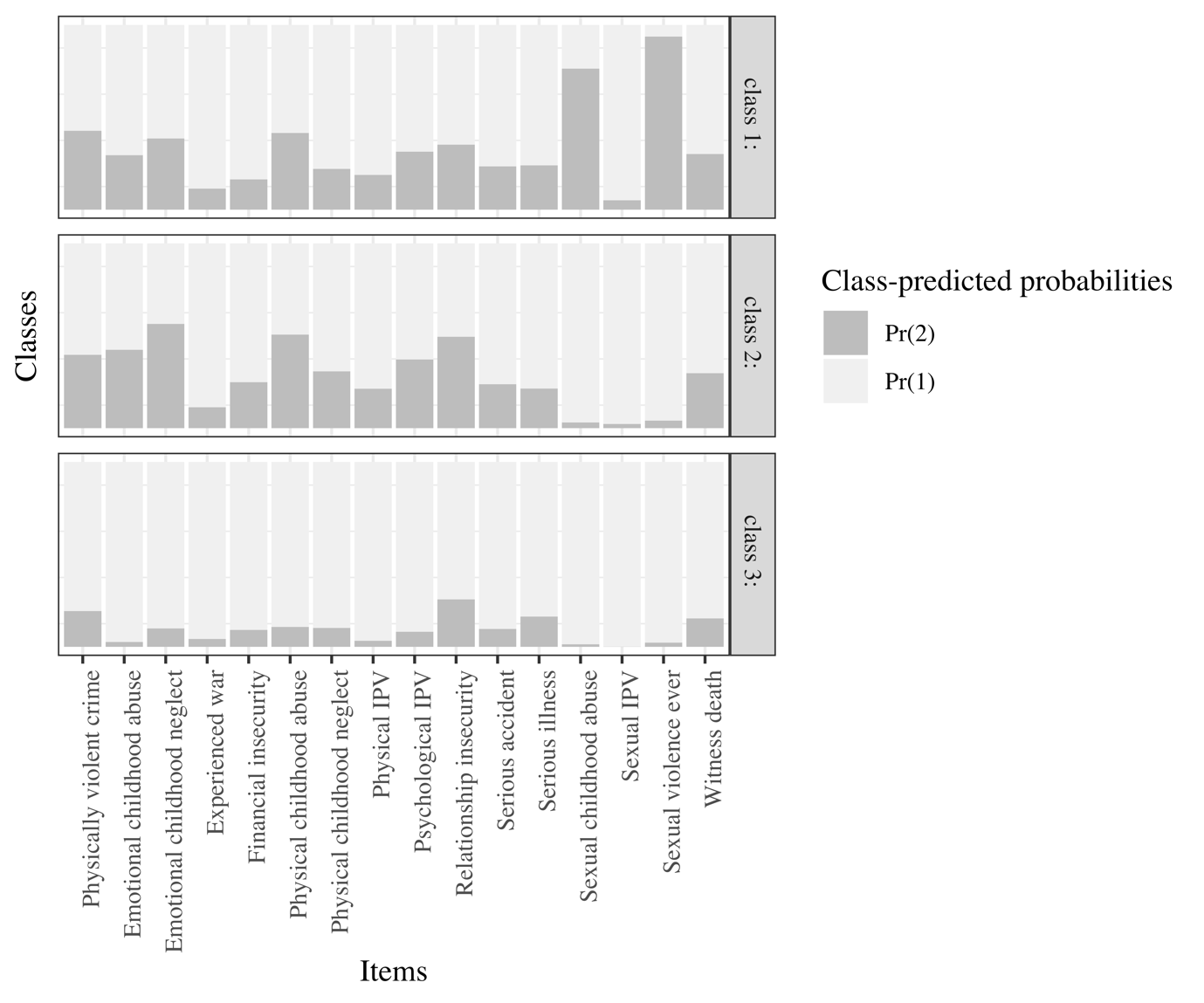


**Figure S4.** 3 class solution in males.

Supplement: Supplementary file 4 — Supplementary file4 (DOCX 179 KB) [file 127_2021_2092_MOESM4_ESM.docx]
