# Supplementary material for: Sex differences in experiences of multiple traumas and mental health problems in the UK Biobank cohort
Source: Soc Psychiatry Psychiatr Epidemiol. 2021 May 10;58(12):1819–31. doi: 10.1007/s00127-021-02092-y (PMC10628045; doi:10.1007/s00127-021-02092-y)
Supplement: Supplementary file 5 — Supplementary file5 (DOCX 14 KB) [file 127_2021_2092_MOESM5_ESM.docx]

|  | **5 class solution** |  |  |  |  | **4 class solution** |  |  |  |
| --- | --- | --- | --- | --- | --- | --- | --- | --- | --- |
| **Class** | **Class 1** | **Class 2** | **Class 3** | **Class 4** | **Class 5** | **Class 1** | **Class 2** | **Class 3** | **Class 4** |
| 1 | 0.91 | 0.02 | 0.03 | 0.04 | 0.00 | 0.76 | 0.12 | 0.06 | 0.05 |
| 2 | 0.07 | 0.79 | 0.05 | 0.05 | 0.04 | 0.03 | 0.94 | 0.02 | 0.02 |
| 3 | 0.05 | 0.04 | 0.82 | 0.04 | 0.04 | 0.04 | 0.06 | 0.84 | 0.07 |
| 4 | 0.11 | 0.03 | 0.04 | 0.78 | 0.03 | 0.04 | 0.05 | 0.06 | 0.85 |
| 5 | 0.00 | 0.04 | 0.07 | 0.06 | 0.83 | - | - | - | - |

**Table S1.** Average posterior probabilities for 5 class and 4 class solutions – females.
