# Supplementary material for: Sex differences in experiences of multiple traumas and mental health problems in the UK Biobank cohort
Source: Soc Psychiatry Psychiatr Epidemiol. 2021 May 10;58(12):1819–31. doi: 10.1007/s00127-021-02092-y (PMC10628045; doi:10.1007/s00127-021-02092-y)
Supplement: Supplementary file 7 — Supplementary file7 (DOCX 15 KB) [file 127_2021_2092_MOESM7_ESM.docx]

|  | **Females (%)** | **Males (%)** |
| --- | --- | --- |
| **Age group** |  |  |
| 45-54 | 14049 (15·8%) | 9429 (13·8%) |
| 55-64 | 31306 (35·1%) | 20567 (30·1%) |
| 65-74 | 37907 (42·5%) | 32227 (47·2%) |
| 75+ | 5835 (6·5%) | 6038 (8·8%) |
| **Ethnicity** |  |  |
| Asian | 599 (0·7%) | 739 (1·1%) |
| Black | 691 (0·8%) | 457 (0·7%) |
| Chinese | 257 (0·3%) | 107 (0·2%) |
| Mixed | 532 (0·6%) | 290 (0·4%) |
| Other | 534 (0·6%) | 343 (0·5%) |
| White | 86179 (96·7%) | 65958 (96·6%) |
| **Townsend Deprivation** |  |  |
| Average | 28325 (31·8%) | 20775 (30·4%) |
| Least | 49544 (55·6%) | 39206 (57·4%) |
| Most | 11120 (12·5%) | 8189 (12·0%) |
| **Household Income** |  |  |
| Less than £18,000 | 12235 (13·7%) | 7338 (10·7%) |
| £18,000-£30,000 | 19296 (21·7%) | 13853 (20·3%) |
| £30,000-£52,000 | 22213 (24·9%) | 18733 (27·4%) |
| £52,000-£100,000 | 18781 (21·1%) | 17926 (26·3%) |
| More than £100,000 | 5561 (6·2%) | 5673 (8·3%) |
| **Mental Health** |  |  |
| Current depression | 1858 (2·1%) | 1049 (1·5%) |
| Current anxiety | 1790 (2.0%) | 889 (1·3%) |
| Current harmful/hazardous alcohol use | 12174 (13.7%) | 20427 (29·9%) |
| Recent psychotic experiences | 418 (0.5%) | 409 (0·6%) |
| **Total** | 89,097 | 68,261 |

**Table S3.** Descriptives of the UK Biobank MHQ stratified by sex.
