# Supplementary material for: Sex differences in experiences of multiple traumas and mental health problems in the UK Biobank cohort
Source: Soc Psychiatry Psychiatr Epidemiol. 2021 May 10;58(12):1819–31. doi: 10.1007/s00127-021-02092-y (PMC10628045; doi:10.1007/s00127-021-02092-y)
Supplement: Supplementary file 8 — Supplementary file8 (DOCX 14 KB) [file 127_2021_2092_MOESM8_ESM.docx]

| Model | Log-likelihood | Residual df | BIC | aBIC | cAIC | Likelihood-ratio | Entropy |
| --- | --- | --- | --- | --- | --- | --- | --- |
| 1 Class | **-1034364.06** | **65519.00** | **2068919.57** | **2068868.72** | **2068935.57** | **202086.92** | **-** |
| 2 Class | **-980906.86** | **65502.00** | **1962208.60** | **1962103.72** | **1962241.60** | **104527.38** | **0.671** |
| 3 Class | **-971419.18** | **65486.00** | **1943436.65** | **1943277.74** | **1943486.65** | **87484.08** | **0.671** |
| 4 Class | -963331.44 | 65468.00 | 1927464.60 | 1927251.68 | 1927531.60 | 72067.78 | 0.705 |
| 5 Class | **-956813.85** | **65451.00** | **1914632.84** | **1914365.89** | **1914716.84** | **60249.15** | **0.678** |
| 6 Class | **-950580.01** | **65434.00** | **1902368.58** | **1902047.60** | **1902469.58** | **48691.30** | **0.677** |

**Table S4.** Fit statistics for latent class models in the whole sample.
